# Control of Expression of Key Cell Cycle Enzymes Drives Cell Line-Specific Functions of CDK7 in Human PDAC Cells

**DOI:** 10.3390/ijms23020812

**Published:** 2022-01-12

**Authors:** Lina Kolloch, Teresa Kreinest, Michael Meisterernst, Andrea Oeckinghaus

**Affiliations:** Institute of Molecular Tumor Biology, Faculty of Medicine, University Muenster, 48149 Muenster, Germany; LinaJenny.Kolloch@ukmuenster.de (L.K.); teresa.kreinest@hotmail.de (T.K.); oeckinga@ukmuenster.de (A.O.)

**Keywords:** cyclin-dependent kinase 7/CDK7, pancreatic cancer, non-covalent CDK7 inhibitor

## Abstract

Inhibition of the dual function cell cycle and transcription kinase CDK7 is known to affect the viability of cancer cells, but the mechanisms underlying cell line-specific growth control remain poorly understood. Here, we employed a previously developed, highly specific small molecule inhibitor that non-covalently blocks ATP binding to CDK7 (LDC4297) to study the mechanisms underlying cell line-specific growth using a panel of genetically heterogeneous human pancreatic tumor lines as model system. Although LDC4297 diminished both transcription rates and CDK T-loop phosphorylation in a comparable manner, some PDAC lines displayed significantly higher sensitivity than others. We focused our analyses on two well-responsive lines (Mia-Paca2 and Panc89) that, however, showed significant differences in their viability upon extended exposure to limiting LDC4297 concentrations. Biochemical and RNAseq analysis revealed striking differences in gene expression and cell cycle control. Especially the downregulation of a group of cell cycle control genes, among them CDK1/2 and CDC25A/C, correlated well to the observed viability differences in Panc89 versus Mia-Paca2 cells. A parallel downregulation of regulatory pathways supported the hypothesis of a feedforward programmatic effect of CDK7 inhibitors, eventually causing hypersensitivity of PDAC lines.

## 1. Introduction

Cyclin-dependent kinases (CDKs) are a family of serine/threonine protein kinases that, together with their associated regulatory cyclins, control central cellular processes. CDKs can generally be divided into kinases that are involved in the molecular control of cell cycle progression (CDKs 1–6, CDKs 14–18) or transcription (CDKs 8–13, CDKs 19–20) [1]. CDK7 holds a special position in this protein family as it functions as a regulator of both processes in vertebrates [2]. As the catalytic core of the CDK-activating kinase (CAK), CDK7 along with cyclinH and Mat1, provides the T-loop phosphorylation and consequent activation of CDK1, CDK2, CDK4 and CDK6, which drive progression through different phases of the cell cycle [3].

In addition, CDK7 plays diverse roles in the regulation of transcription. As a component of the general transcription factor II human (TFIIH), CDK7 phosphorylates Ser-5 of the carboxy-terminal domain (CTD) of RNA polymerase II (RNAPII) to facilitate promotor escape and transcription initiation [4,5,6]. CDK7 also phosphorylates and activates CDK9 to in turn phosphorylate Ser-2 in the CTD of RNAPII, a process that is required for pause release and productive transcription elongation [7]. In vitro assays suggested that CDK7 is involved in mRNA capping and the promotion of pausing through facilitating the association of DRB-sensitivity inducing factor (DSIF) and negative elongation factor (NELF) with RNAPII [8,9]. Furthermore, CDK7-mediated phosphorylation directly regulates the activity of various transcription factors, including retinoic acid receptor [10,11,12], androgen receptor [13,14], oestrogen receptor [15,16], and p53 [17,18]. Components of the splicing machinery have also been identified as targets of CDK7-dependent phosphorylation and widespread mRNA splicing defects have been observed upon inhibition of CDK7 [19].

Due to its dual role in proliferation and transcription, CDK7 represents an especially intriguing target amongst the CDKs for potential therapeutic strategies in oncology. An aberrant increase of CDK7 levels has been detected in many different cancer types, e.g., gastric, pancreatic, colorectal and breast cancer, and often correlates with aggressiveness and poor prognosis [20,21,22,23]. Knockdown or CRISPR/Cas9-mediated knockout of CDK7 results in reduced cell proliferation of gastric cancer and triple-negative breast cancer cells, respectively, highlighting a functional role of CDK7 in tumor cell growth [21,22]. In addition, THZ1, a covalent inhibitor of CDK7, reduces viability of a broad range of cancer cell lines from different entities, including thyroid carcinoma, lung squamous cell carcinoma, cervical cancer, colorectal cancer and pancreatic cancer, with T-cell acute lymphoblastic leukemia cells being especially sensitive [23,24,25,26,27]. THZ1 has also been shown to be effective in MYCN-driven neuroblastoma and small cell lung cancer cells, where treatment showed preferential inhibition of superenhancer-driven genes including MYCN and other oncogenes, suggesting that CDK7 inhibition might be an interesting therapeutic strategy for transcription-addicted and MYC-driven cancers [28,29].

The fact that THZ1 also targets CDK12 and CDK13 at concentrations used to affect CDK7 activity has, however, complicated a clear attribution of cellular phenotypes to CDK7 inhibition and fueled the search for more selective CDK7 inhibitors [30]. Several other CDK7 inhibitors have now been reported in the literature, four of which, ICEC0942, SY-1365, SY-5609 and LY340515 have progressed to Phase I/II clinical trials (for recent comprehensive reviews see [31,32]). We previously reported a triazine class of ATP-competitive CDK7 inhibitors, LDC4297 and LDC3140, with especially high specificity for CDK7 [33]. LDC4297 is the lead substance within the triazine class, selected for and based on both high affinity to the ATP binding site of CDK7 in the lower nanomolar range and specificity for CDK7. Testing LDC4297 in in vitro kinase assays revealed high selectivity for CDK7 in the CDK group and no reactivity to a panel of 150 non-related kinases [33,34]. Our previous studies using these inhibitors demonstrated control of mRNA synthesis, cell cycle progression and survival by CDK7 in different tumor cell lines [33]. LDC4297 blocks RNA polymerase II transcription rates with virtually identical IC50 values in mammalian extracts and inside cells at physiological ATP levels ([33] and unpublished data).

The progression of healthy cells to pancreatic ductal adenocarcinoma (PDAC) requires the accumulation of several genetic mutations [35]. More than 90% of human pancreatic tumors harbor genetic changes in the *KRAS* gene, resulting in constitutively active KRAS and consequent proliferative signaling [36]. Additional frequently occurring mutations target the *CDKN2A* (cyclin-dependent kinase inhibitor 2A; 80–95% of cases), *TP53* (50% of cases) or *SMAD4* (50–60% of cases) loci [37]. The *CDKN2A* gene encodes for the tumor suppressors INK4 and ARF (ADP-ribosylation factor). INK4 acts as an inhibitor for CDK4 and CDK6 in the cell cycle, while ARF stabilizes p53 through inhibition of MDM2-dependent proteolysis [38,39,40]. The transcription factor p53 is involved in cell-cycle arrest, apoptosis, senescence, metabolism, and DNA repair [41]. SMAD4 is an effector of TGFβ-induced signaling, which plays an important role in pancreatic cancer development [42,43,44]. Beyond these main driver mutations, PDAC reveals great heterogeneity with different genetic profiles and (secondary) mutations associated with differences in clinical manifestations and response to therapeutic approaches, which poses a challenge to finding treatment options suitable for a majority of PDAC patients [45].

Here, we studied the mechanisms underlying cell-specific responses to limited CDK7 inhibition using a panel of PDAC lines and LDC4297 as a highly potent and specific kinase inhibitor. Specific focus was on two representative cell lines, Mia-Paca2 and Panc89, originating from different stages and tissues of human pancreatic tumors and reacting to CDK7 inhibition with distinct sensitivity. Under the conditions of limited inhibition, we observed a marked impact on transcription rates, while T-loop phosphorylation of CDKs was moderately influenced, none of which, however, accounted for differential sensitivity of PDAC lines. RNAseq and protein analyses—following long-term inhibition with limiting concentrations of the CDK7 inhibitor—eventually revealed broad regulation of expression of cell cycle genes. The latter correlated well to both downregulation of cell cycle activity and differential sensitivity of PDAC lines in viability assays. Further linked to sensitivity, expression of the critical cell cycle regulators E2F1 and NFY declined over days. Our findings provide a mechanistic explanation for cooperative downregulation of cell cycle genes, illuminating the basis for novel programmatic roles of CDK7 in cancer cells.

## 2. Results

### 2.1. Broad Response but Selective Sensitivity of PDAC Lines to Inhibition of CDK7

**Figure 1:**
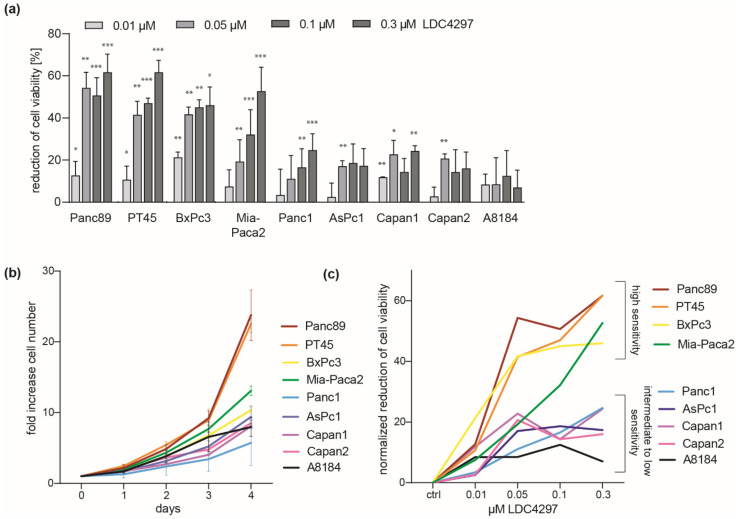
**PDAC cells display cell line-specific sensitivity towards CDK7 inhibition.** (**a**) Different PDAC cell lines were treated with the indicated doses of LDC4297 for 3 days and viability was measured. Shown is mean ± SD (*n* = 3, exceptions: Panc89: 0.1 and 0.3 µM *n* = 6, Mia-Paca2: 0.05 *n* = 11, 0.1 µM *n* = 18 and 0.3 µM *n* = 13, Panc1: 0.1 µM *n* = 8 and 0.3 µM *n* = 8). Significance was analyzed by two-sided Student’s *t* test: * *p* ≤ 0.05; ** *p* ≤ 0.01; *** *p* ≤ 0.001. (**b**) PDAC cell proliferation was followed over the course of 4 days. Shown is mean ± SEM (*n* = 3). (**c**) Mean values of measurements from (**a**) were used to normalize the effects of LDC4297 treatment on all cell lines to their respective proliferation rate (Supplementary Table S2). Graph shows the reduction of viable cells for each LDC4297 concentration after normalization to the respective proliferation rate of each cell line.

A representative panel of nine human pancreatic cancer cell lines was chosen to investigate the effects of non-covalent CDK7 inhibition by LDC4297 (Supplementary Table S1). The panel reflected the different mutational states of key oncogenic alterations in PDAC and included cells derived from the main tumor body as well as from metastases. We treated the PDAC cell lines with increasing concentrations of LDC4297 up to 0.3 µM and analyzed cell viability in assays using ATP levels as a measure. CDK7 inhibition effectively reduced the number of viable Panc89, PT45 and BxPc3 cells already at a concentration of 0.05 µM LDC4297 identifying these cell lines as a “good responder” group (Figure 1a). Mia-Paca2 cells showed intermediate to good sensitivity, with a reduced response at low LDC4297 concentrations < 0.1 µM. Panc1, Capan1, Capan2, AsPc1 and A8184 cells were less sensitive, with A8184 cells displaying the lowest sensitivity to CDK7 inhibition.

Differences in sensitivity between cell lines did not obviously relate to the main genetic modifications involved in the transformation of PDAC cells (Supplementary Table S1). As one prominent feature, the data suggested that strong impact of CDK7 inhibition did not depend on a mutated Ras gene. For example, the wildtype KRas-expressing line BxPc3 qualified for the “good responder” group, as do the mutant KRas-expressing lines Mia-Paca2 and PT45. Further, in support of a correlation to the proliferation status, analysis of the slow growing human fibroblast line MRC5 revealed a moderate and more linear response to LDC4297, reasoning for a correlation to proliferation rates (Supplementary Figure S1a). Indeed, when we measured proliferation and determined respective doubling times all good responder PDAC lines belonged to the faster proliferating cells (Figure 1b and Supplementary Table S2). However, differences in the sensitivity towards LDC4297 between cell lines persisted after normalization to the determined doubling times, reasoning for further mechanisms mediating the dependence on CDK7 (Figure 1c).

### 2.2. CDK7 Affects All Cell Cycle Phases with Preference for G1/S in Panc89

**Figure 2:**
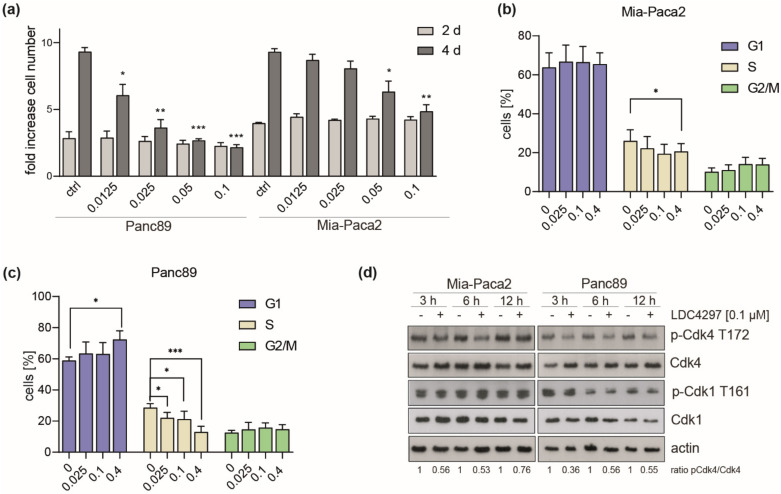
**Differential alterations in cell cycle distribution following CDK7 inhibition.** (**a**) Mia-Paca2 and Panc89 cells were treated with the indicated concentrations of LDC4297 [µM] for 2 and 4 days and viability was analyzed. Shown are the mean ± SD (*n* = 3). (**b**,**c**) Mia-Paca2 (**b**) and Panc89 (**c**) cells were treated with the indicated LDC4297 concentrations [µM] for 4 days and cell cycle states analyzed by FACS. Shown are the mean ± SD (*n* = 5). (**d**) Immunoblot analysis of Mia-Paca2 and Panc89 lines treated with 0.1 µM LDC4297 for 3, 6 or 12 h. Intensities of bands were analyzed by Image Studio Lite software (Licor). Significance was analyzed by two-sided Student’s *t*-test: * *p* ≤ 0.05; ** *p* ≤ 0.01; *** *p* ≤ 0.001.

We next studied the effect of LDC4297 treatment on cell cycle progression. Being primarily interested in the mechanisms causing strong CDK7 dependency, we chose to focus on the comparison of one intermediate (Mia-Paca2) and one good responder (Panc89/T3M-4) cell line. A specific reason for the choice of these cell lines was that they differed in sensitivity at low LDC4297 concentrations (below 0.1 µM, Figure 2a). Consequently, FACS analysis was conducted in the concentration range ≤ 0.4 µM LDC4297. Both cell lines responded with a marked reduction in S-phase populations (Figure 2b,c). A G1-arrest was observed for Panc89 at high (0.4 µM) LDC4297 concentrations (Figure 2c). However, S-phase populations already declined significantly in the range of hypersensitivity, reasoning for an arrest in G1/S- and/or G2/M phase for Panc89, whereas Mia-Paca2 cells required higher LDC4297 concentrations to show significant changes (Figure 2b).

We next asked whether sensitivity differences between the two lines related to the established CAK function of CDK7 using CDK4 as primary target. Indeed, T-loop phosphorylation by CDK7 had not been investigated neither for our inhibitor class nor, to our knowledge, generally in PDAC lines. Following treatment with LDC4297 for 3 to 12 h, T-loop phosphate levels of CDK4 were reduced in both lines (Figure 2d). The observed changes in the two cell lines were comparable, reasoning against a role in differential response to the CDK7 inhibitor. Within this time frame we observed little impact on CDK1-Thr161-P, which required longer exposure to the inhibitor, possibly because the T-loop of CDK1 is better protected against dephosphorylation (Supplementary Figure S1b) [46,47]. We also measured changes in apoptosis rates following CDK7 inhibition. These were comparable in Mia-Paca2 and Panc89 and altogether too low to explain the strong impact on viability in both lines (Supplementary Figure S1c).

### 2.3. Evidence for a Concentration-Dependent Crosstalk between CDK7 and CDK4

**Figure 3:**
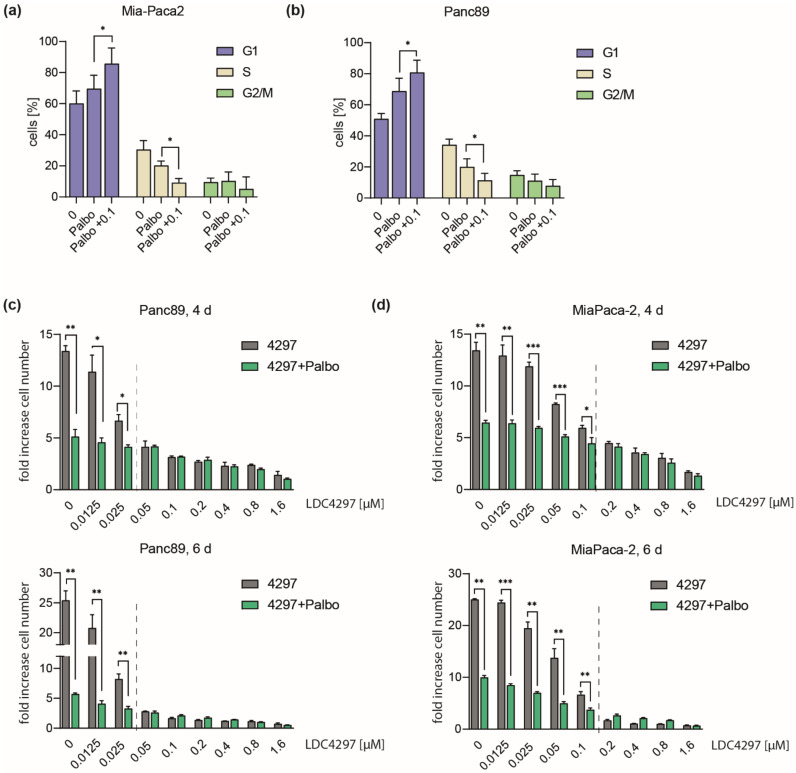
**Concentration-dependent interplay of Palbociclib with LDC4297.** (**a**,**b**) Mia-Paca2 and Panc89 cells were treated with 2 µM Palbociclib in the absence or presence of 0.1 µM LDC4297 for 4 days, DAPI stained and analyzed by FACS. Shown is the mean ± SD (*n* = 4). (**c**,**d**) Panc89 and Mia-Paca2 cells were treated with the indicated concentrations of LDC4297 [µM] in the absence or presence of 2 µM Palbociclib. Cell viability was analyzed after 4 and 6 days. Shown is the mean ± SD (*n* = 3). Dashed line indicates the border of the (sub-)additivity range of CDK7 and CDK4 inhibitors. Significance was analyzed by two-sided Student’s *t* test: * *p* ≤ 0.05; ** *p* ≤ 0.01; *** *p* ≤ 0.001.

Next, we became interested in a potential cooperation of CDK7 with cell cycle checkpoint kinases. Here, we characterized CDK4 which is activated in many cancer cells and against which a potent and specific inhibitor (Palbociclib) is available. Not unexpected, Palbociclib induced a marked increase of the G1 population in both Mia-Paca2 and Panc89 lines (Figure 3a,b). Co-treatment of cells with Palbociclib and LDC4297 further enriched G1 populations, reaching 80–85%, while the G2/M- and most prominently S-phase populations declined in parallel. This was seen for both Mia-Paca2 and Panc89 cells (Figure 3a,b).

In the proliferation analysis, co-treatment with Palbociclib further reduced growth in the low LDC4297 concentration range (0.025–0.1 µM) (Figure 3c,d). However, above a threshold LDC4297 concentration (dashed line in Figure 3c,d), Palbociclib remained without additional effect. Threshold borders essentially matched concentrations of LDC4297 at which proliferation ceased in the individual lines (below 0.05 µM for Panc89 and 0.2 µM for Mia-Paca2, compare Figure 2a). Above threshold concentrations, the CDK7 inhibition alone apparently fully overruled the impact of cell cycle kinases. Such a take-over by LDC4297 above threshold levels was also seen when we combined LDC4297 with the CDK1/2 inhibitor Ro-3306. However, different from CDK4, CDK1 and CDK7 additivity remained minimal at low LDC4297 concentrations (Supplementary Figure S2). Mechanistically, our data reason for a cooperation of CDK7 and CDK4 on G1/S arrest in both Mia-Paca2 and Panc89 cells. This is likely explained by blocking CDK4 activity from two angles by simultaneously inhibiting ATP binding (Palbociclib) and limiting T-loop phosphorylation (LDC4297). It thus seems conceivable that cooperation requires limiting concentrations of inhibitors. This may also open potential treatment perspectives for cancer lines that have elevated CDK4 activities and show high proliferation rates.

### 2.4. Comparable Impact of CDK7 Inhibition on Transcription in Mia-Paca2 and Panc89 Cells

**Figure 4:**
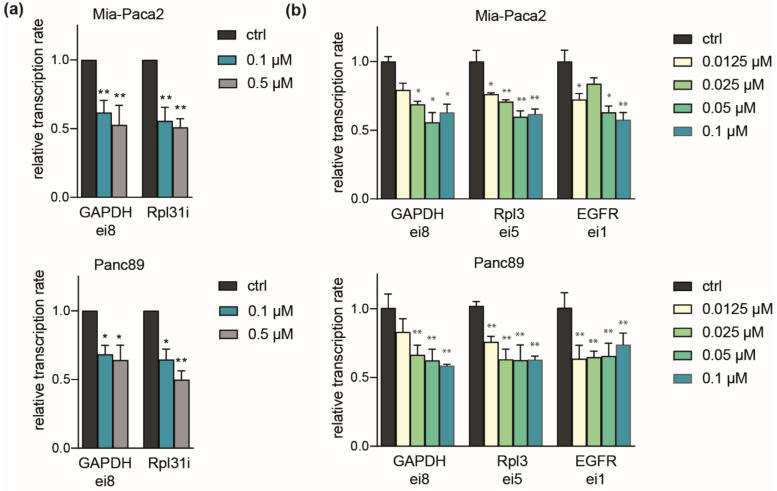
**Comparable impact of CDK7 on transcription in Mia-Paca2 and Panc89 cells.** (**a**) Mia-Paca2 and Panc89 cells were treated with 0.1 µM and 0.5 µM LDC4297 for 2 h and transcription rates determined by RT-qPCR using primers targeting intronic RNA directly (i) or RNA at exon-junctions (ei). Results were normalized to spike-in RNA (Luciferase). Shown is the mean ± SD (*n* = 4). (**b**) Mia-Paca2 and Panc89 cells were treated with the indicated concentrations of LDC4297 [µM] for 2 h and analyzed as described in (**a**). Shown is the mean ± SD (*n* = 4). Significance was analyzed by two-sided Student’s *t* test: * *p* ≤ 0.05; ** *p* ≤ 0.01.

We further asked whether differential responses of PDAC lines related to an influence on gene transcription. To measure levels of nascent RNA, we made use of the short half-life of unspliced intronic RNA, which was analyzed in RT-qPCR using specific intronic (i) or exon-intron (ei) junction primers [33]. Validated primers against housekeeping targets (*GAPDH* and *Rpl31*) revealed a marked (50%) drop in transcription rates after 2 h exposure at 0.1 to 0.5 µM LDC4297. Importantly, transcription rates for both genes were reduced similarly in Mia-Paca2 and Panc89 cells, reasoning against a general difference in transcriptional impact of CDK7 inhibition between the two cell lines (Figure 4a). This was also not the case at low concentrations (starting from 12.5 nM) where Mia-Paca2 and Panc89 differ in viability (Figure 4b), but which suffice to cause noticeable decreases in transcription. Further noteworthy, the first intron of the EGFR (epidermal growth factor receptor), which has been annotated as a superenhancer-driven gene in PDAC [48,49] reacted similarly at the transcriptional level to GAPDH and Rpl3. These data confirm the role of CDK7 in RNAP-II transcription without revealing differences associated with individual cell line sensitivity.

### 2.5. Prolonged Limited Inhibition of CDK7 Leads to Activation of NF-κB Target Genes

**Figure 5:**
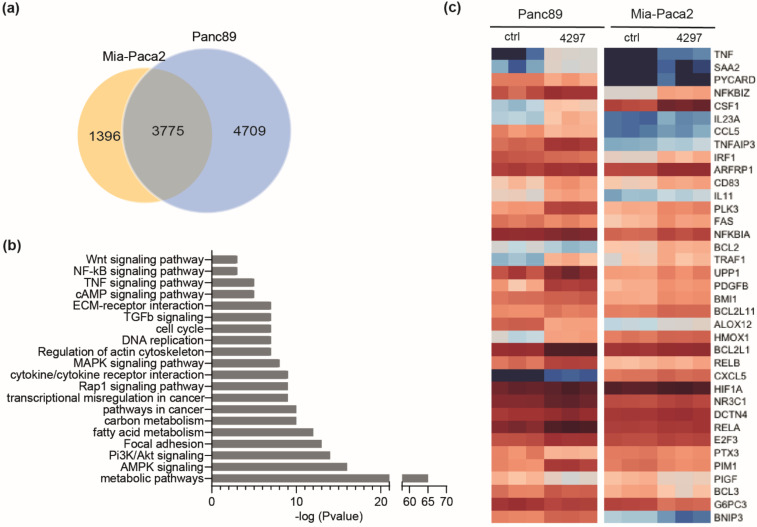
**Prolonged limited inhibition of CDK7 leads to activation of NF-κB target genes.** (**a**) Venn diagram representing number of genes deregulated in Panc89 and Mia-Paca2 cells after treatment with 0.1 µM LDC4297 for 4 days as determined by RNAseq analysis. (**b**) KEGG pathway analysis of genes deregulated in both Panc89 and Mia-Paca2 cells by treatment with 0.1 µM LDC4297 for 3 days. (**c**) Heatmap of NF-κB target genes, which are significantly regulated in Panc89 and Mia-Paca2 cells in RNAseq analysis, sorted according to regulation in Mia-Paca2. Upregulation: 76% in Panc89, 84% in Mia-Paca2. Downregulation: 24% in Panc89, 16% in Mia-Paca2.

Next, RNAseq analysis was conducted with Mia-Paca2 and Panc89 cells. These were treated for three days with 0.1 µM LDC4297, a concentration that elicited both robust effects and showed differences between the two cell lines (compare Figure 2a). CDK7 inhibition significantly altered the expression of 8484 genes in Panc89 and of 5171 genes in Mia-Paca2 cells (Figure 5a). A total of 3775 genes (44% of all genes found altered in their expression) were deregulated in both cell lines, indicating gross overlap but also significant differences in the control of gene expression. Validity of the array was confirmed with RT-qPCR analyses of several upregulated and downregulated genes (Supplementary Figure S3a,b and Supplementary Table S3). The KEGG pathway analysis of the core signature genes regulated in both cell lines identified metabolic pathways as the most prominently and largely negatively affected processes. In addition, cell cycle and diverse signaling pathways were altered with high significance (Figure 5b and Supplementary KEGG analysis). Interestingly, several of the latter pathways seemed activated in response to extended limited CDK7 inhibition. Among them were the TGFβ (Supplementary Figure S3c) and NF-κB pathways. NF-κB target genes were activated in both Panc89 and Mia-Paca2 cells (Figure 5c). This was unexpected given that NF-κB had been characterized as a negatively responding activator. However, these data were restricted to an early response to the covalent CDK7 inhibitor THZ-1 [23]. Whether our observation reflects a complementary activation remains under investigation. Preliminary data indicated that the effect on cell viability remained too low to accelerate Mia-Paca2 cell growth sufficiently to explain the differences between the two cell lines (unpublished data). We hence decided to search for other pathways in the RNAseq data set.

### 2.6. CDK7 Inhibition Caused Downregulation of Myc in PDAC Lines

**Figure 6:**
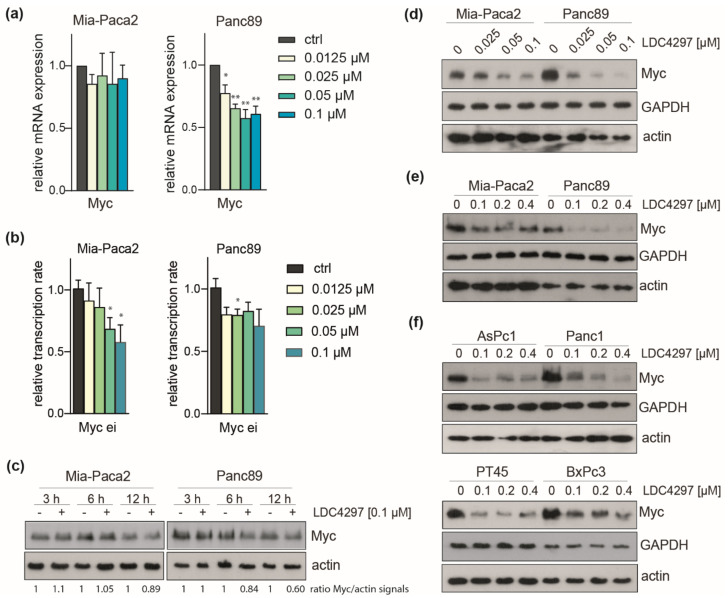
**CDK7 inhibition causes downregulation of Myc in PDAC lines.** (**a**) Analysis of Myc mRNA expression by RT-qPCR in Panc89 and Mia-Paca2 cells treated with the indicated concentrations of LDC4297 for 3 days. Shown is the mean ± SD (*n* = 4). (**b**) Analysis of Myc transcription rate by RT-qPCR using Myc ei primers in Mia-Paca2 and Panc89 cells treated with the indicated concentrations of LDC4297 for 2 h. Shown is the mean ± SD (*n* = 3). (**c**) Immunoblot analysis of Myc protein level in Mia-Paca2 and Panc89 cells treated with 0.1 µM LDC4297 for the indicated times. (**d**,**e**) Immunoblot analysis of Myc protein level in Mia-Paca2 and Panc89 cells treated with the indicated concentrations of LDC4297 for 4 days. (**f**) Immunoblot analysis of Myc protein level in AsPc1, Panc1, PT45 and BxPc3 cells treated with the indicated concentrations of LDC4297 for 4 days. Significance was analyzed by two-sided Student’s *t* test: * *p* ≤ 0.05; ** *p* ≤ 0.01.

Examination of the Myc protooncogene, a key inducer and effector gene in cancer and established player in PDAC [23], showed moderate (20%) downregulation in Panc89 and no response in Mia-Paca2 cells in our RNAseq data. This was confirmed with RT-qPCR. The decline of Myc mRNA was fully established at low (0.0125 and 0.025 µM) concentrations of LDC4297 in Panc89 cells, whereas Mia-Paca2 cells remained unresponsive up to 0.1 µM LDC4297 (Figure 6a). In contrast, the Myc transcription rate was efficiently downregulated in both lines 2 h after addition of the inhibitor (Figure 6b), indicating that Myc gene expression is subject to control by the respective cellular programs. Downregulation of the Myc protein was detectable starting at around 6 h after addition of the inhibitor in Panc89 cells (Figure 6c). Furthermore, after treatment with LDC4297 for 4 days, the impact of CDK7 inhibition in the low concentration range was less pronounced in Mia-Paca2 cells (Figure 6d). Myc protein levels were also analyzed after 4 days treatment with higher LDC4297 concentration (0.1–0.4 µM range). Again, Panc89 reacted stronger than Mia-Paca2 cells, showing full response at the lowest (0.1 µM) inhibitor concentration (Figure 6e). We concluded that the performance of Myc correlated qualitatively with higher sensitivity of Panc89 cells.

We subsequently asked whether Myc is also differentially regulated in other PDAC lines. This relates to the question whether Myc control critically determines the viability properties within the initially defined “good and poor” responder groups. In brief, Myc responded to the inhibitor in all lines tested (AsPc1 and Panc1 as members of the low responder group and PT45 and BxPc3 as good responders) and this response was at least as good as the one seen in Mia-Paca2 cells (Figure 6f). Furthermore, one member of the “good” (PT45) and one of the “poor” responder group (Panc1) displayed significant concentration dependency. In sum, the data suggest that Myc is regulated in all PDAC lines without, however, showing a strict correlation to the proliferation rates of groups and individual lines in response to CDK7 inhibition.

### 2.7. Evidence for Broad Control of the Expression of Key Cell Cycle Control Genes by CDK7

**Figure 7:**
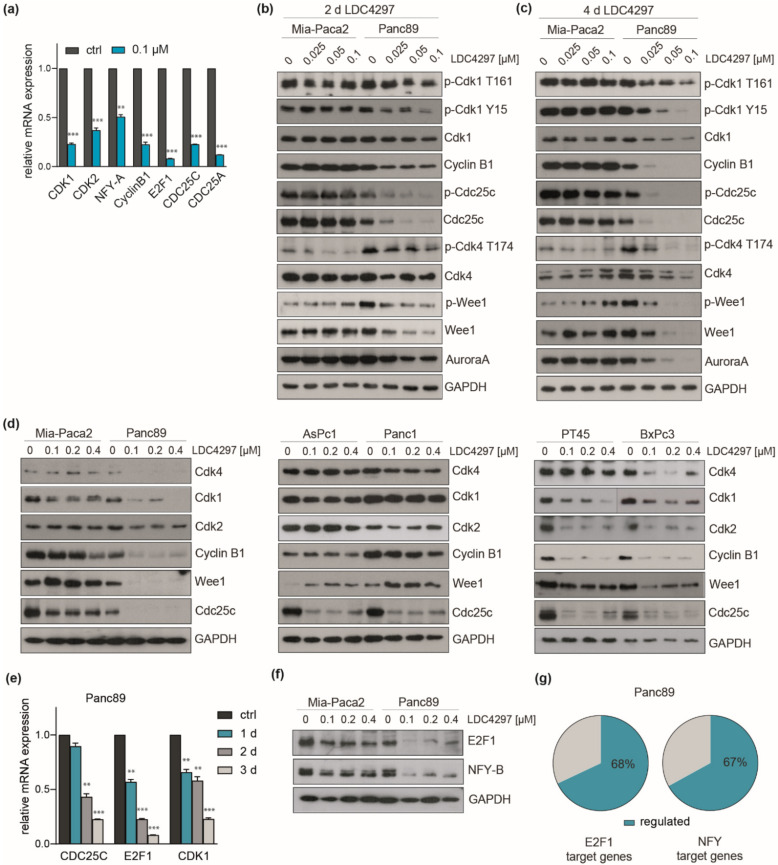
**Cell line-specificity of cell cycle regulator RNA and proteins.** (**a**) RT-qPCR analysis of Panc89 RNA after treatment with 0.1 µM LDC4297 for 3 days. Shown is the mean ± SD (*n* = 3). (**b**,**c**) Western blot analysis of Mia-Paca2 and Panc89 cells treated for 2 days (**b**) or 4 days (**c**) with the indicated antibodies. (**d**) Western blot analysis of PDAC lines after treatment for 4 days with 0.1, 0.2 and 0.4 µM LDC4297. (**e**) RT-qPCR analysis of representative cell cycle control genes in Panc89 RNA after treatment for 1, 2 and 3 days with 0.1 µM LDC4297. Shown is the mean ± SD (*n* = 3). (**f**) Western blot analysis of E2F1 and NFY-B after treatment of Panc89 and Mia-Paca2 cells for 4 days with the indicated concentrations of LDC4297. (**g**) Schematic representation of E2F1 and NFY target genes regulated by LDC4297 treatment in Panc89 cells taken from RNAseq analysis. Significance was analyzed by two-sided Student’s *t* test: ** *p* ≤ 0.01; *** *p* ≤ 0.001.

Our next focus was on genes related to the cell cycle machinery. A closer manual inspection of RNAseq data eventually revealed broad regulation of cell cycle control genes. These included the upregulation of cell cycle inhibitor genes (CDKN1A (p21), 1C (p57), 2A (p16), 2B (p15)). Furthermore, the expression of critical cell cycle kinases CDK1, CDK2, CDK4 and WEE1 (G2 checkpoint kinase), CDK-tyrosine phosphatases CDC25A and CDC25C (cell cycle division 25A und C), CyclinB1 and many other genes related also to S-phase control/DNA replication dropped significantly (Table 1 and Supplementary Figure S4a). Representative members of the group were validated with RT-qPCR (Figure 7a). Importantly, the decline throughout the group (listed in Table 1) was stronger in Panc89 than in Mia-Paca2 cells, reasoning for a link to hypersensitivity of Panc89.

Immunoblots conducted with Mia-Paca2 and Panc89 cell lysates lent further support to this hypothesis. CDK1, CDK4 and WEE1 as well as CyclinB1 were increasingly and selectively downregulated over time in Panc89 cells at limiting LDC4297 concentrations (2 days in Figure 7b and 4 days in Figure 7c). The decline of CyclinB1 was clearly detectable at day 2 at the lowest (0.025 µM) inhibitor concentration in Panc89 but not in Mia-Paca2 cells. At day 4, CyclinB1 was still not altered in Mia-Paca2 while it was undetectable in Panc89 lysates. The latter observation fits well with the observed selective G1/S arrest in Panc89 (Figure 2).

The T-loop phosphorylation status of CDKs following longer exposure to CDK7 inhibitor (2 versus 4 days, Figure 7b,c, respectively) was essentially similar to the one described in the short-term analysis (Figure 2d) if normalized to the decline in kinase expression (Supplementary Figure S4b). In addition, Tyrosine 15 phosphorylation of CDK1 was markedly downregulated in Panc89 cells. In parallel, the responsible kinase WEE1 is downregulated too, illuminating a potential link within the cell cycle block. CDC25C was also dramatically downregulated, again selectively in Panc89 (Figure 7b).

We next investigated the impact of LDC4297 treatment in this respect on a broader panel of PDAC lines. To be able to see effects in poor responders we now chose an intermediate concentration range of LDC4297 (0.1–0.4 µM). Importantly, the two good responders BxPc3 and PT45 showed marked downregulation of CDK1, CDK2, CyclinB1, WEE1 and less strict also of CDK4. In contrast, the poor responders AsPc1 and Panc1 responded moderately, thereby more closely resembling Mia-Paca2 cells (Figure 7d). In the end, the impact on the cell cycle machinery in Mia-Paca2 cells was rather mild relative to the intermediate to good proliferation sensitivity profile of this cell line, suggesting that here further processes/factors might mediate CDK7 inhibition. Individual heterogeneity is also seen in the small set of factors analyzed here. For example, CDC25C was also regulated in all lines and did not consistently align with the proliferation response.

The E2F family and the CCAAT box binding complex NFY are critical regulators of cell cycle genes, among them CDK1, CDK2 and CDC25C [50,51,52,53,54]. Both regulatory factors were downregulated in RNAseq (Table 1) with E2F-1 and NFY-A, -B subunits being among the strongest responders. A time course RT-qPCR analysis with E2F-1, CDC25C and CDK1 as read-out reasoned for a delayed time-dependent, non-linear decay of the respective mRNAs (Figure 7e) with little effect seen after one day of treatment. This is generally consistent with both the protein analysis as well as proliferation/viability data. Further consistent with our hypothesis, E2F1 and NFY proteins were strongly downregulated in Panc89 and much less in Mia-Paca2 cells (Figure 7f). Finally, target gene analysis revealed that 68% of the annotated E2F1 target genes and 67% of the NFY target genes were deregulated in Panc89 cells in our RNAseq data (Figure 7g).

Collectively, our study discloses a striking correlation of sensitivity towards CDK7 inhibition with the broad regulation of gene expression of cell cycle-associated genes between the cell lines Mia-Paca2 and Panc89 that could well explain the sensitivity differences seen for these cell lines at low inhibitor concentrations. This includes downregulation of critical drivers of the cell cycle and of relevant regulator families, as well as upregulation of CDK inhibitors, with the latter possibly being related to the parallel downregulation of Myc. Several critical cell cycle genes and the Myc protein were also regulated in a larger panel of PDAC lines, reasoning for a general importance. The concomitant transcription regulation of both—cell cycle genes and relevant regulatory factors—may well establish a novel programmatic basis for proliferation control of human tumor cells by CDK7.

## 3. Discussion

The importance of CDK7 for proliferation of cancer cells is intimately linked to activation of CDKs (CAK function), which is setting the pace in cell cycle progression, as well as to an essential role during pausing and early elongation of RNA polymerase II [46,55,56]. Beyond this, examples for an impact on specific regulatory pathways and control of specific genes have been reported. Overall, however, the basis of selectivity for defined cancer cells remains not well understood at present. Our investigations of a panel of human pancreatic tumor cells now reason for a broad role of CDK7 in maintaining expression of genes involved in control and execution of the cell cycle program. Downregulation of the cell cycle program rather than differences in CDK T-loop phosphorylation (CAK) activity or general transcription provided explanations for cell-specific sensitivity to a highly selective CDK7 inhibitor characterized previously [33,34]. In fact, all PDAC lines tested here respond to CDK7 inhibition but show significant differences in their sensitivity. Downregulation of key players in cell cycle control such as CDK1, CDK2, CDK4 and CyclinB1, as well as relevant cell cycle gene regulators NF-Y [50] and E2F family members [57] correlate especially well with sensitivity differences in the lines Mia-Paca2 and Panc89. We also report evidence for further programmatic alterations, among them activation of the NF-κB pathway, compensatory in nature, that appear to determine the response of this genetically highly diverse group of cancer cells to CDK7 inhibition.

Cancer studies on CDK7 gained momentum upon discovery of the covalently binding inhibitor THZ1 that blocks growth of various cancer entities, among them triple negative breast cancer, Myc-associated tumors, non-small cell lung carcinoma and also pancreatic cancer [23,58]. In several studies, including investigations in PDAC lines, THZ1 action was mechanistically linked to suppression of lineage-determining genes that are under the control of large enhancer elements (so called superenhancers, SE) [23,24,25,28,29]. When we looked at the pancreatic SE genes, we could not observe coherent suppression at the mRNA level (Supplementary Figure S4c). Furthermore, suppression of the transcription rates of the SE genes, Myc and EGFR, was comparable to GAPDH and other house-keeping genes. Discrepancies between inhibitors may well be due to the known cross-reactivity of THZ1 with CDK12 and CDK13, facilitating a more comprehensive response of RNAPII transcription [32,58]. Our inhibitor class in turn is highly selective for CDK7, showing no inhibition of CDK12 and CDK13 [33,34]. Moreover, the excellent specificity of the LDC4297-underlying core structure was recently further underlined by novel structurally related compounds (unpublished data). Several other covalently and non-covalently binding inhibitors have been described to be effective in cancer cells [31,32]. In one study, using the inhibitor SY-351, CDK7 activity was linked to broad phosphorylation of the splicing machinery, raising the question whether there is a direct effect of CDK7 on splicing [19]. Notably, our observations that are based on qPCR analysis of hnRNA (exon-intron primer analysis, Figure 4) presently do not reason for a general positive function of CDK7 in splicing. In fact, LDC4297 acts generally negative on hnRNA levels, whereas bona-fide splice inhibitors dramatically enhance the half-life of intronic RNA (data not shown).

A second PDAC investigation, conducted with THZ1 while this investigation was ongoing, emphasized the influence of transcription repression especially of NF-κB target genes as a possibly pioneering event in CDK7 inhibition [23]. Surprisingly, our long-term observations suggest the opposite, namely moderate but broad activation of NF-κB target genes. This is seen in both cell lines investigated at the RNAseq level, Mia-Paca2 and Panc89. Consistent with the RNAseq data, we do observe upregulation of IKKβ-dependent, p65-mediated NF-κB activity (unpublished results). However, also in our setting NF-κB target genes were suppressed after short exposure to LDC4297 (data not shown). We have not studied the reversal of the process in vitro that, however, might be of interest in the highly inflammatory environment of pancreatic tumors [59].

Another potentially compensatory mechanism indicated by the RNAseq data relates to the TGFβ pathway. The TGFβ ligands TGFβ1 and TGFβ2 are upregulated as are the corresponding receptor genes upon LDC4297 treatment (Supplementary Figure S3c). Different to activation of the NF-κB pathway, regulation of TGFβ genes is specific for the Panc89 cell line. One negatively regulated target gene in the tumor-suppressive pathway of TGFβ is the Myc protooncogene. Myc is indeed specifically downregulated in Panc89, while CDKN1A (p21) and CDKN2B (p15), both subject to repression by Myc, are upregulated (Table 1). However, inhibitors against the TGFβ1 (ALK5) receptor failed to show a significant impact, neither on Panc89 proliferation nor on expression of Myc (data not shown). We thus assume that Myc is controlled by other processes and TGFβ itself, while being a candidate to influence transformation in vivo, has little impact on proliferation in vitro.

Myc levels in turn are broadly correlated with the repression by CDK7 in PDAC lines. This is an important effector pathway of our inhibitor class given that Myc has been described as a critical factor for PDAC formation in vivo [60,61]. Myc is indeed a direct, sensitive CDK7-dependent target, which is likely in part conferred by the short half-life of both mRNA and protein [33,62]. Genes encoding mRNA and proteins with low stability like Myc are in fact good candidates to initiate a broader suppressive process. Our data also indirectly reason for control of protein stability of Myc. Myc mRNA dropped 1.3-fold in Panc89, whereas Myc protein levels virtually disappeared after longer exposure to LDC4297. Perhaps related to this, we do see upregulation of the Myc ubiquitin-conjugating enzyme E2, as well as ubiquitin ligase Trim32 and Myc-destabilizing GSK3-β, while the Myc-stabilizing ligases Usp28, Sirt2 and β-Trc are downregulated after extended inhibition [63]. Much of our investigation focused on resolving sensitivity differences for the two lines Mia-Paca2 and Panc89. Myc control correlates very well with differences in these two lines, while it is less predictive for the response in the broader PDAC panel. In fact, Mia-Paca2 itself is somewhat exceptional in that it showed the least Myc response within this panel. This, however, was correlated with limited upregulation of potential Myc ubiquitin ligases as cited above.

The perhaps most striking observation of this study, the broad regulation of cell cycle genes, has been developed from the Mia-Paca2–Panc89 comparison. Here, expression regulation remained generally correlative to LDC4297 sensitivity, at least for certain key genes like CDK1, CDK2 and cyclin B1 within the broader PDAC panel. Of note, individual cell cycle genes have been identified as CDK7-dependent in recent studies based on genetic knockdown and non-covalent inhibitor studies. Impact on E2F-controlled genes after ablation of CDK7 or inhibition with the inhibitor YKL-5-124 has been reported previously although without further follow-up studies [64,65]. Additional examples for cell cycle genes shown to be under CDK7 control include CDK1 and Aurora kinases [66]. Of note, Aurora kinases have also been linked to Myc stabilization [67]. Our data shows that CDK7 inhibition also significantly impacted expression of the CCAAT-binding transcription complex NF-Y, which is known to play important roles in the regulation of cell cycle genes and proliferation [50]. E2F1 has been described as an NF-Y target gene; however, other studies describe E2F1-dependent transcription of NF-Y [50,51,68]. Cyclin B1, CDK1, CDK4, CDC25A and CDC25C are NF-Y target genes [50,51]. Our study demonstrates that the proliferation response of PDAC lines to CDK7 inhibition correlates well to the kinetics of the parallel downregulation of cell cycle machinery and regulatory factor genes, which together with effects on T-loop phosphorylation and general transcription provides the basis for feedforward downregulation of cell cycle target genes. The observed specific G1 cell cycle arrest of Panc89 in turn relates well to downregulation of, i.e., CDK2 and CDK4. Furthermore, NF-Y knockdown has been reported to result in a G1 cell cycle arrest [51]. NF-Y transcription factors are known for their roles in various types of cancer including renal cell carcinoma, breast cancer, gliomas and colorectal adenocarcinomas [51,69,70,71]. However, to our knowledge this is the first report on a potential cancer-promoting involvement of NF-Y-dependent transcription in pancreatic cancer.

We have not yet extended the full RNA and protein analysis to the broader panel of PDAC lines. Ongoing preliminary RT-qPCR studies investigating the second strongest responder cell line PT45, however, initially confirm the observation of a broad control of cell cycle genes including E2F1 and also NF-Y subunits (data not shown). Already the selected protein analysis lends support to the hypothesis of a more general role of cell cycle gene expression control by CDK7 in pancreatic cancer. Potential therapeutic applications will certainly rely on the use of limiting CDK7 inhibitor concentrations as applied here in this study. Thereby, our non-covalently binding inhibitor class also offers options for combinatorial treatments, as exemplified here for the CDK4 inhibitor Palbociclib. It will now be important to further validate both the relevance of our findings and the applicability of selected inhibitors [72] within this structural class in animal cancer models.

## 4. Materials and Methods

### 4.1. Cell Culture and Inhibitors

PDAC cell lines (Panc89, PT45, Mia-Paca2, BxPc3, Panc1, Capan1, AsPc1, Capan2, A8184) were maintained in RPMI-1640 supplemented with 10% heat-inactivated FBS (fetal bovine serum) and antibiotics (penicillin/streptomycin) at 37 °C in a 5% CO_2_ incubator. Cells were passaged every 2–3 days using 0.25% trypsin. MRC5 cells were cultured in MEM supplemented with 10% FBS and antibiotics, and HEK293FT cells in DMEM containing 10% FBS, antibiotics and 1% L-glutamine. The following inhibitors were used: LDC4297 (MedChemExprees, Monmouth Junction, NJ, USA; HY-12653), Ro-3306 (MedChemExprees, HY-12529) and Palbociclib (MedChemExprees, HY-50767), TGFbi (BIBF0775, MedChemExprees, HY-13783), Galunisertib (MedChemExprees, HY-13226).

### 4.2. Cell Viability Assay

300–1000 cells per well were seeded in white, tissue culture-treated 96-well-plates and treated with inhibitors 24 h later. After the indicated time periods, CellTiter-Glo^®^ Reagent (Promega, Madison, WI, USA) was added directly to the cells and luminescence was measured according to manufacturer’s instructions using a Berthold plate reader.

### 4.3. FACS Analyses

Cells were cultured as described above and treated with inhibitors for the indicated times. Medium plus inhibitor was refreshed at day 3 of treatment. To perform FACS analyses, cells were harvested by trypsinization. For cell cycle analyses, 100 µL of cell suspension were diluted with 900µL DAPI solution (3 µM; Applichem, Darmstadt, Germany) and cells stained for 15 min at room temperature. Cells were then analyzed by FACS. For analysis of apoptosis rates, cells were stained with an Apoptose Staining Kit AnnexinV-FITC (BD Pharmingen, San Diego, CA, USA #55654) according to manufacturer’s instructions. The results were evaluated using the FlowJo software.

### 4.4. Cell Lysis and Immunoblotting

Whole cell extracts were generated using RIPA lysis buffer (200 mM NaCl, 20 mM Tris-HCL pH 7.3, 10% glycerol, 1 mM EDTA (pH 8), 0.5% sodium deoxycholate, 0.1% SDS, 1% IGEPAL) supplemented with phosphatase- and protease inhibitors and 1 mM DTT/15 mM β-Mercapoethanol. Protein concentrations were determined using Bradford-Reagent (Sigma-Aldrich, St. Louis, MO, USA) and adjusted accordingly. Proteins were separated by SDS-PAGE and transferred onto PVDF membranes using a semidry blotting system. After blocking with Odyssey blocking buffer (LI-COR Bioscience, Lincoln, NE, USA) diluted 1:1 with PBS or 5% milk/TBST, membranes were probed with primary antibodies at a dilution of 1:1000 overnight. Protein bands were visualized on a Licor Odyssey CLx system (Image Studio Software version 5.2 LI-COR) or using ECL (Thermo Fisher Scientific, Waltham, MA, USA). The following antibodies were used: α-Tubulin (Santa Cruz, Dallas, TX, USA; sc-23948), Myc (Cell Signaling Technology, Danvers, MA, USA, 18583), pCDK1 Thr161 (Cell Signaling Technology, 9114), CDK1 (Cell Signaling Technology, 77055), pCDK4 Thr172 (Abclonal, Woburn, MA, USA; AP0593), CDK4 (Santa Cruz, sc-56277), CDK2 (Santa Cruz, sc-6248), GAPDH (Proteintech, Planegg, Germany, 60004), Actin (Santa Cruz, sc-1615), Wee1 (Santa Cruz, sc-5285), E2F1 (Santa Cruz, sc-251), pCDC25C S216 (Cell Signaling Technology, 4901), NFY-B (Santa Cruz, sc-376546) and CyclinB1 (Cell Signaling Technology, 4138). All primary antibodies were diluted 1:1000. Secondary antibodies were purchased from LI-COR: IRDye^®^800CW (α-mouse), IRDye^®^680RD (α-rabbit) or Jackson ImmunoResearch, West Grove PA, USA: anti-mouse-HRP (115-035-044) and anti-rabbit-HRP (111-035-045).

### 4.5. RNA-seq Analysis

Total RNA was prepared from TRIzol (Life Technologies, Carlsbad, CA, USA) lysates. Briefly, 1 ml Trizol samples were mixed with 200 µL chloroform, incubated for 3 min and then centrifuged for 15 min at 12,000× *g* at 4 °C. The aqueous phase was transferred to a fresh tube and mixed with 0.5 ml isopropanol, followed by 10 min incubation and centrifugation for 10 min at 12,000× *g* at 4 °C. The pellet was washed twice with 75% ethanol, centrifuging at 7500× *g* for 5 min at 4 °C. Pellets were finally air-dried and dissolved in RNAse-free water. RNA integrity was assessed using the Agilent Bioanalyzer RNA Nano (RIN value at least 8.5). Libraries were prepared via NEBNext mRNA enrichment and the NEBNext Ultra RNA Prep Kit. Quality of the libraries was controlled via Agilent Bioanalyzer HighSens DNA Chip followed by NEBNext qPCR library quantification and equimolar pooling. Samples were sequenced using an Illumina NextSeq500 system (v2 chemistry). For analysis the R package DESeq2 was used as previously described [73]. As a reference hg38 genome was used. Transcripts with a DESeq2 FDR-adjusted *p*-value < 5% were categorized as significant.

### 4.6. RNA Extraction and RT-qPCR

For additional gene expression analyses, cells were lysed in TRIzol reagent and RNA isolated as described above for RNAseq analysis. 500–1000 ng RNA was used for reverse transcription (Thermo Fisher, Revert Aid). For analyses addressing transcription rates, RNA was extracted using the Direct-zol RNA Mini Prep kit (Zymo Research, Irvine, CA, USA; R2050) including a DNA digest step and cDNA was generated with the PrimeScript RT Kit with gDNA eraser (Takara, Shiga, Japan; RR047A) according to manufacturer’s instructions. qPCRs were performed using SYBR Green (NEB, Ipswich, MA, USA; Luna SYBR Master) on a StepOnePlus Real-Time PCR System (Thermo Fisher). For data collection and analysis, a 7300 System SDS Software version 2.3 was used. RT-qPCR Primers were as follows (Table 2). Primers that are labeled as “ei” generate products that cross exon/intron borders and thus allow determination of transcription rates. For primers that are labeled “i” both primers for the RT-qPCR align with intronic sequences also allowing for determination of transcription rates.

### 4.7. Statistics

Statistical analysis was conducted using Prism Software (GraphPad Prism version 8). Unless stated otherwise, all n numbers represent independently performed experiments, and statistical tests are two-sided, unpaired TTESTs. Where representative experiments are shown, experiments have been performed at least two times independently.

## Figures and Tables

**Table 1:** **RNAseq changes of selected genes connected to cell cycle control** (n.a. not altered, n.e. not expressed).

Symbol	Panc89	MP2
	Log2FC	Log2FC
CDKN1A (p21)	2.00	0.66
CDKN1B (p27)	−0.59	n.a.
CDKN1C (p57)	1.38	0.15
CDKN2A (p16)	1.11	n.e.
CDKN2B (p15)	2.78	n.e.
CDKN2C (p18)	−2.43	0.60
CDK1	−1.57	−0.60
CDK2	−1.02	−0.53
CDK4	−1.12	n.a.
CDK7	0.74	n.a.
CCNA1	−0.90	n.e.
CCNA2	−1.85	−0.62
CCNB1	−1.51	−0.30
CCNB2	−1.15	−0.44
CCND1	0.62	0.50
CCND3	0.53	n.a.
CDC25A	−1.19	n.a.
CDC25B	−1.46	−0.26
CDC25C	−2.05	−0.58
CHEK1	−0.95	−0.52
CHEK2	−1.81	−1.20
WEE1	−1.45	0.53
E2F1	−1.90	−0.83
E2F2	−1.52	−1.09
E2F4	−0.30	−0.41
E2F5	−0.55	n.a.
E2F7	−0.59	n.a.
E2F8	−1.71	n.a.
NFYA	−0.65	n.a.
NFYB	−0.99	n.a.
NFYC	0.59	0.19
RBL1	−1.48	−0.75
RBL2	−0.69	−0.31
TRAP1	−1.50	−0.68
AURKA	−1.83	−0.43
AURKB	−1.70	−0.43
AURKC	2.70	1.31

**Table 2:** Primers.

Primer	Sequences
hActin_F	GCTGTGCTGTCCCTGTATGCCTCT
hActin_R	CCTCTCAGCTGTGGTGGTGAAGC
hBcl-xL_F	AGGAGAACGGCGGCTGGGATA
hBcl-xL_R	GAGCCCAGCAGAACCACGCC
hCDC25C_1F	TCTACGGAACTCTTCTCATCCAC
hCDC25C_1R	TCCAGGAGCAGGTTTAACATTTT
hCDC25A_1F	TTCCTCTTTTTACACCCCAGTCA
hCDC25A_1R	TCGGTTGTCAAGGTTTGTAGTTC
hCDCP1_F	CTGAACTGCGGGGTCTCTATC
hCDCP1_R	GTCCCCAGCTTTATGAGAACTG
hCDK1_1F	AAACTACAGGTCAAGTGGTAGCC
hCDK1_1R	TCCTGCATAAGCACATCCTGA
hCDK2_1F	CCAGGAGTTACTTCTATGCCTGA
hCDK2_1R	TTCATCCAGGGGAGGTACAAC
hCyclinB1_1F	AATAAGGCGAAGATCAACATGGC
hCyclinB1_1R	TTTGTTACCAATGTCCCCAAGAG
hEGFR_F	AACTGTGAGGTGGTCCTTGG
hEGFR_R	TGAGGACATAACCAGCCACC
hEGFRei1_F	AGGGCGTCATCAGTTTCTCA
hEGFRei1_R	AGTTCTCCTCTCCTGCACCC
hEIFD2_F	GCCTTTCGGGTCAAGTCCAA
hEIFD2_R	CCTCCTTTCCAGGTACTAACTCA
hGAPDHei8_F	GCCCTGACAACTCTTTTCATCT
hGAPDHei8_R	TCTCTCTTCCTCTTGTGCTCTTG
hMYBL2_F	CCGGAGCAGAGGGATAGCA
hMYBL2_R	CAGTGCGGTTAGGGAAGTGG
hMYC_F	TTTCGGGTAGTGGAAAACCA
hMYC_R	CACCGAGTCGTAGTCGAGGT
hMYCei1_F	TAACTCAAGACTGCCTCCCG
hMYCei1_R	AAGCTAACGTTGAGGGGCAT
hNFY-A_1F	ATGTGGTCAATTCAGGAGGGA
hNFY-A_1R	ATTGTTTGGCATTCACGTAGAGA
hPRPF3_F	CAGCAGCATTGAACTGTGTGG
hPRPF3_R	TCGTCGCTTCTTTACTCCTGAT
hRPL3ei5_F	CAAGGGCAAAGGCTACAAAG
hRPL3ei5_R	GAATGGTTCTACACTGTCCGATT
hRPL31i1_F	TTTGGGATTGAACTGG
hRPL31i1_R	CCCTAAGCCTACTTTC
hTAF15_F	GATTCTGGAAGTTACGGTCAGTC
hTAF15_R	AGCTTTGTGATGCTTGTCCATAG
hTNFRSF10D_F	TACCACGACCAGAGACACC
hTNFRSF10D_R	CACCCTGTTCTACACGTCCG
hTRAF6_F	ATG CGG CCA TAG GTT CTG C
hTRAF6_R	TCCTCAAGATGTCTCAGTTCCAT

## Data Availability

RNAseq data generated for this manuscript will be provided upon request.

## Supplementary Materials

The following are available online at https://www.mdpi.com/article/10.3390/ijms23020812/s1, Supplementary Files and tables are provided as a combined pdf file and KEGG pathway analysis as EXCEL file.
