# Supplementary material for: Control of Expression of Key Cell Cycle Enzymes Drives Cell Line-Specific Functions of CDK7 in Human PDAC Cells
Source: Int J Mol Sci. 2022 Jan 12;23(2):812. doi: 10.3390/ijms23020812 (PMC8775745; doi:10.3390/ijms23020812)
Supplement: Supplementary file 1 [file ijms-23-00812-s001.zip › ijms-1504584-supplementary-revised.pdf]

Supplementary Figures

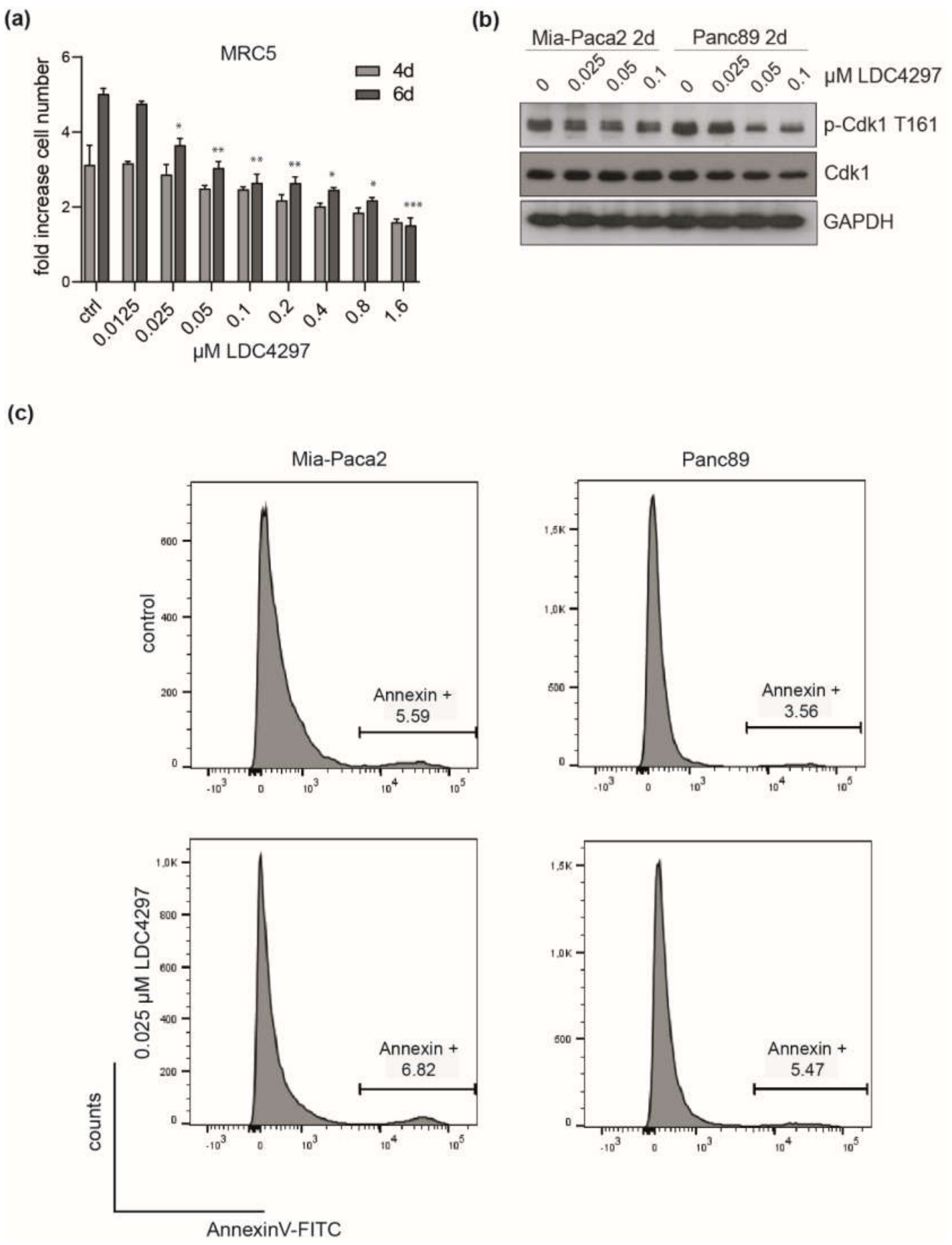

Supplementary Figure S1: (**a**) MRC5 cells were treated with the indicated concentrations of LDC4297 [µM] for 4 or 6 days and cell viability analyzed (n = 3). Significance was analyzed by two-sided Student's t test: * $p \le 0.05$; ** $p \le 0.01$; *** $p \le 0.001$. (**b**) Mia-Paca2 and Panc89 cells were treated with the indicated concentrations of LDC4297 [µM] or DMSO for 48 hours. Cell extracts were analyzed by SDS-PAGE and immunoblot using the indicated antibodies. (**c**) Mia-Paca2 and Panc89 cells were treated with 0,025 µM LDC4297 for 4 days. Cells were stained with Annexin V-FITC and analyzed by FACS.

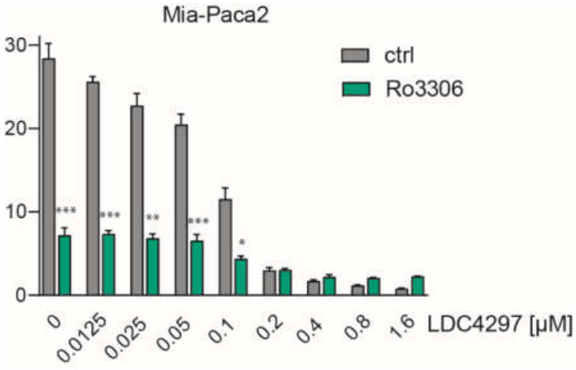

Supplementary Figure S2: Mia-Paca2 cells were treated with increasing concentrations of LDC4297 [μM] in the absence or presence of 2 μM Ro-3306 and cell viability determined (n = 3). Significance was analyzed by two-sided Student's t test: * p≤0.05; ** p≤0.01; *** p≤0.001

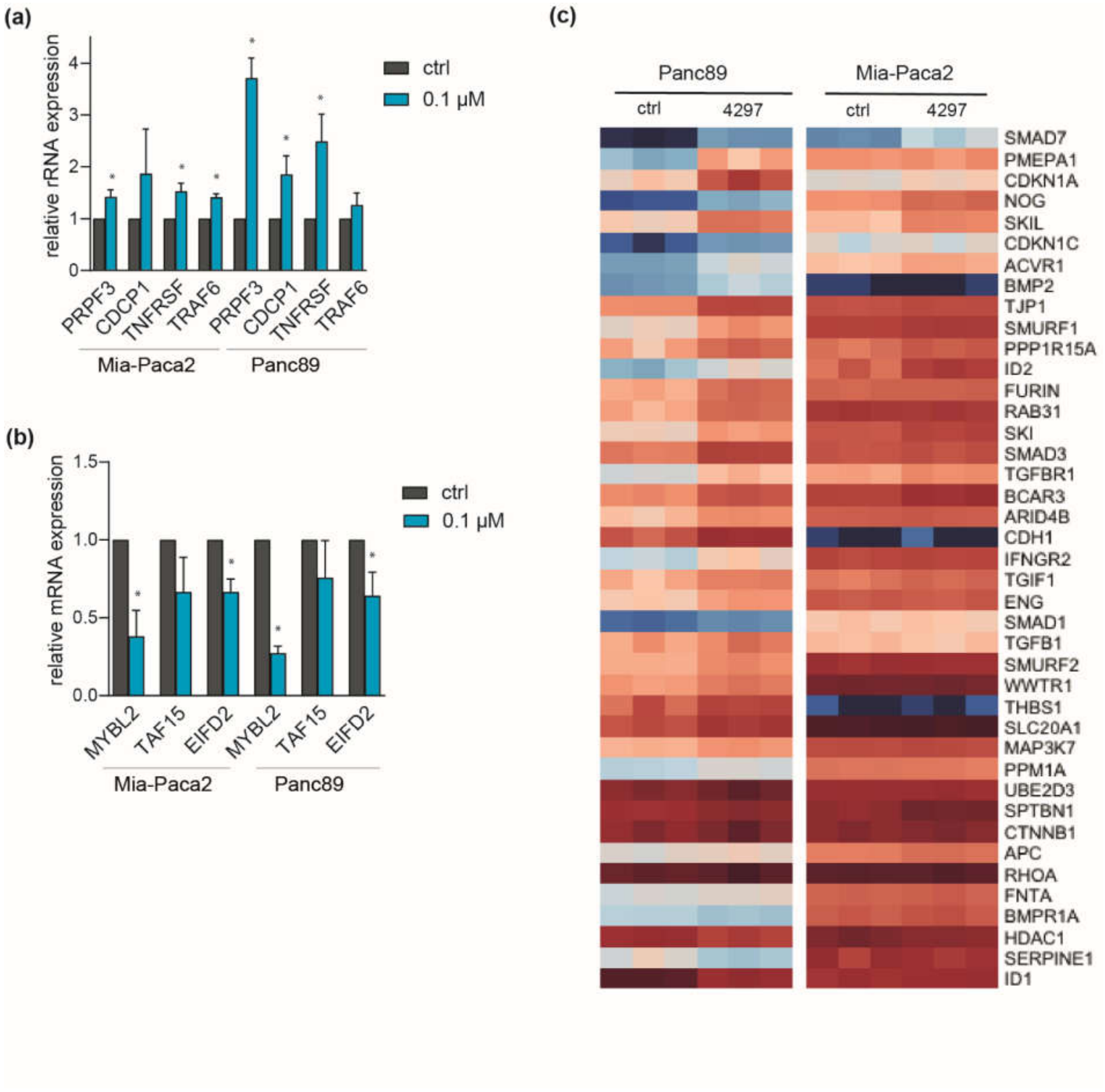

Supplementary Figure S3: (**a, b**) Mia-Paca2 and Panc89 cells were treated with 0.1 μM LDC4297 for 3 days and mRNA expression of exemplarily chosen genes either found upregulated (a) or downregulated (b) in RNAseq analysis was analyzed by RT-qPCR (n = 2). (**c**) Heatmap of TGFβ target genes from RNAseq data, which are significantly regulated in Panc89, sorted according to regulation in Panc89. Upregulation: 90% in Panc89, 37% in Mia-Paca2; Downregulation: 10% in Panc89, 2% in Mia-Paca2; 61% not significantly regulated in Mia-Paca2.

Significance was analyzed by two-sided Student's t test: * $p \leq 0.05$; ** $p \leq 0.01$; *** $p \leq 0.001$

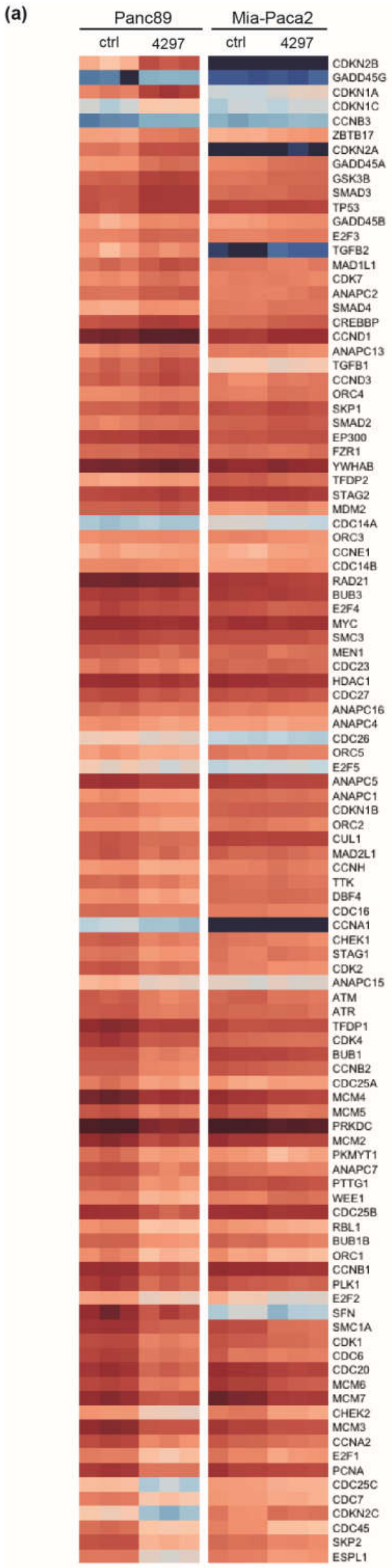

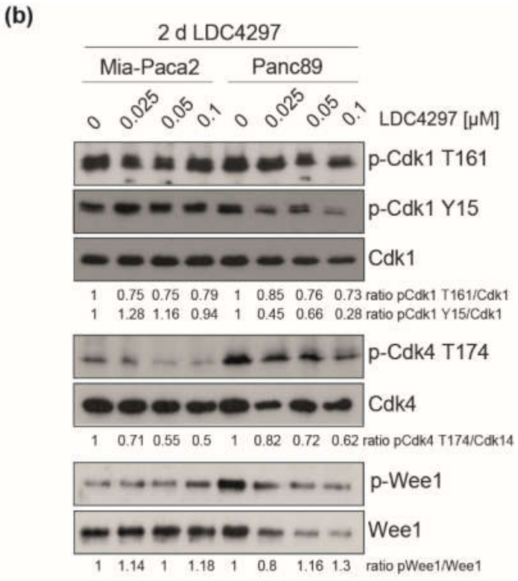

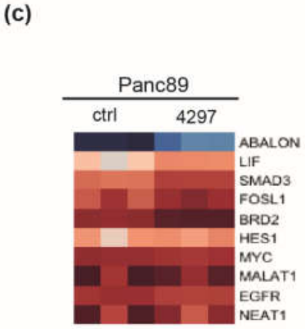

Supplementary Figure S4: (**a**) Heatmap of cell cycle related genes (KEGG cell cycle pathway), which are significantly regulated in at least one of the cell lines, sorted according to regulation in Panc89. Upregulation: 29.5% in Panc89, 17.1% in Mia-Paca2; Downregulation: 65.7% in Panc89, 44.8% in Mia-Paca2; 4.8% not significantly regulated in Panc89, 38.1% not significantly regulated in Mia-Paca2. (**b**) Immunoblots of extracts from Mia-Paca2 and Panc89 cells treated with LDC4297 for 2 days. Band intensities were quantified using ImageJ software. (**c**) Heatmap depicting the expression level of genes that were reported to be driven by superenhancers in PDAC cells in Panc89 cells with and without treatment with 0.1 µM LDC4297 for 3 days.

Supplementary Tables

Suppplementary Table S1. Mutational status of K-Ras, p53, CDKN2A and Smad4 in human PDAC cell lines.

	Cell lines								
**Locus**	Panc89 [1]	PT45 [2]	BxPc3 [3]	MP2 [4]	Panc1 [5]	AsPc1 [6]	Capan1 [7]	Capan2 [7]	A8184 [2]
KRas	Q61H	G13D	wt	G12C	G12D	G12D	G12V	G12V	G12R
p53	Y220C	R280K	Y220C	R248W	R273H/ R273C	R273H/ 135 frameshift	A159V	wt/ R273H	mut. in tetra-merization domain
CDKN2A	mut. meth.	mut. h.d.	wt	mut. h.d.	mut. h.d.	wt/ frame-shift	mut. h.d.	wt./ mut. ins.	mut. h.d.
Smad4	wt	wt	mut. h.d.	wt	wt	wt/ R100T	F557L	wt	wt
**Tissue origin**	lymphnode metastasis	primary tumor	primary tumor	primary tumor	primary tumor	ascites/ metastatic site	liver metastasis	primary tumor	ascites

Abbreviations: wt: wild-type, mut.: mutation, meth.: methylation, h.d.: homozygous deletion, ins.: insertion. Cell lines are sorted from left to right by decreasing sensitivity to LDC4297.

Supplementary Table S2. Proliferation rates of human PDAC cell lines.

	Cell lines								
	Panc89	PT45	BxPc3	Mia-Paca2	Panc1	AsPc1	Capan1	Capan2	A8184
doubling time [d]	0.899	0.8233	1.087	0.9942	1.722	1.262	1.378	1.140	1.139

Supplementary Table S3: Log2 fold changes determined from RNAseq analysis and RT-qPCR for the indicated genes

symbol	Panc89 Log2FoldChange		MiaPaca2 Log2FoldChange	
	**RNAseq**	**qPCR**	**RNAseq**	**qPCR**
PRPF3	1.92	4.69	0.76	1.42
CDCP1	2.12	1.85	1.20	1.86
TNFRSF10D	2.19	2.49	0.89	1.53
TRAF6	1.03	1.26	0.54	1.41
MYBL2	-2.58	-0.27	-1.59	-0.38
TAF15	-2.28	-0.76	-1.16	-0.66
EIF2D	-2.24	-0.64	-1.69	-0.67

Supplementary References

[1] T. Okabe, N. Yamaguchi, and N. Ohsawa, "Establishment and characterization of a carcinoembryonic antigen (CEA)-producing cell line from a human carcinoma of the exocrine pancreas.," *Cancer*, vol. 51, no. 4, pp. 662–8, Feb. 1983.

[2] P. S. Moore *et al.*, "Genetic profile of 22 pancreatic carcinoma cell lines. Analysis of K-ras, p53, p16 and DPC4/Smad4.," *Virchows Arch.*, vol. 439, no. 6, pp. 798–802, Dec. 2001.

[3] M. H. Tan *et al.*, "Characterization of a new primary human pancreatic tumor line.," *Cancer Invest.*, vol. 4, no. 1, pp. 15–23, 1986.

[4] A. A. Yunis, G. K. Arimura, and D. J. Russin, "Human pancreatic carcinoma (MIA PaCa-2) in continuous culture: sensitivity to asparaginase.," *Int. J. cancer*, vol. 19, no. 1, pp. 128–35, Jan. 1977.

[5] M. Lieber, J. Mazzetta, W. Nelson-Rees, M. Kaplan, and G. Todaro, "Establishment of a continuous tumor-cell line (panc-1) from a human carcinoma of the exocrine pancreas.," *Int. J. cancer*, vol. 15, no. 5, pp. 741–7, May 1975.

[6] W. H. Chen *et al.*, "Human pancreatic adenocarcinoma: in vitro and in vivo morphology of a new tumor line established from ascites.," *In Vitro*, vol. 18, no. 1, pp. 24–34, Jan. 1982.

[7] B. Sipos, S. Möser, H. Kalthoff, V. Török, M. Löhr, and G. Klöppel, "A comprehensive characterization of pancreatic ductal carcinoma cell lines: towards the establishment of an in vitro research platform," *Virchows Arch.*, vol. 442, no. 5, pp. 444–452, May 2003.
